# Current-Voltage Characteristics of Membranes with Different Cation-Exchanger Content in Mineral Salt—Neutral Amino Acid Solutions under Electrodialysis

**DOI:** 10.3390/membranes12111092

**Published:** 2022-11-02

**Authors:** Vera I. Vasil’eva, Elmara M. Akberova, Ali M. Saud, Victor I. Zabolotsky

**Affiliations:** 1 Department of Analytical Chemistry, Chemical Faculty, Voronezh State University, Universitetskaya pl. 1, 394018 Voronezh, Russia; 2 Faculty of Science, Tishreen University, Latakia 2237, Syria; 3 Department of Physical Chemistry, Faculty of Chemistry and High Technologies, Kuban State University, ul. Stavropolskaya 149, 350040 Krasnodar, Russia

**Keywords:** cation-exchange membrane, resin content, ampholyte, water splitting, current-voltage characteristic, limiting current

## Abstract

The features of the electrochemical behavior of experimental heterogeneous ion-exchange membranes with different mass fractions of sulfonated cation-exchange resin (from 45 to 65 wt%) have been studied by voltammetry during electrodialysis. Electromembrane systems with 0.01 M NaCl solution and with a mixed 0.01 M NaCl + 0.05 M phenylalanine (Phe) solution have been investigated. A significant influence of the ion-exchanger content on the parameters of current-voltage curves (CVCs) was established for the first time. Electrodialysis of the sodium chloride solution revealed a decrease in the length of the limiting current plateau and in the resistances of the second and third sections of the CVCs with an increase in the resin content in the membrane. The fact of the specific shape of the CVCs of all studied cation-exchange membrane samples in mixed solutions of the mineral salt and the amino acid was established. A specific feature of current-voltage curves is the presence of two plateaus of the limiting current and two values of the limiting current, respectively. This phenomenon in electromembrane systems with neutral amino acids has not been found before. The value of the first limiting current is determined by cations of the mineral salt, which are the main current carriers in the system. The presence of the second plateau and the corresponding second limiting current is due to the appearance of additional carriers due to the ability of phenylalanine as an organic ampholyte to participate in protolytic reactions. In the cation-exchange electromembrane system with the phenylalanine containing solution, two mechanisms of H^+^/OH^−^ ion generation through water splitting and acid dissociation are shown. The possibility of the generation of H^+^/OH^−^ ions at the enriched solution/cation-exchange membrane interface during electrodialysis of amino acid containing solutions is shown for the first time. The results of this study can be used to improve the process of electromembrane demineralization of neutral amino acid solutions by both targeted selection or the creation of new membranes and the selection of effective current operating modes.

## 1. Introduction

The ability of ampholytes (amino acids, acid salts of polybasic organic and inorganic acids, proteins, hydrolysis products of alkaline, etc.) to be present in the solutions in various ionic forms or neutral molecules depending on the solution pH determines the electrochemical behavior of ion-exchange membranes in electromembrane systems. These forms (anions, bipolar ions or neutral molecules) are involved in protonation-deprotonation reactions with ions of the medium [1,2,3,4,5], water [6,7,8,9] and counterions in membranes [7,10,11]. When an electric current is applied to the electromembrane system, the concentration of current carriers at the membrane surface decreases to values comparable to the concentration of protons (hydroxyl ions) formed in aqueous solutions because of water splitting. Under such conditions, usually rapid protonation-deprotonation reactions of ampholytes can determine the rate of the overall process of electromass transfer [12]. The model calculations show that, owing to the protonation reaction, the flux of glycine amino acid can increase by 30% [11].

The ability of amino acids (organic ampholytes) to hydrolyze with the generation of H^+^ and OH^−^ ions and, depending on solution pH, to transform into various ionic forms is apparently the main reason for the difference in electrochemical characteristics of ion-exchange membranes in amino acid containing solutions in comparison with solutions of strong electrolytes [3,13,14]. In particular, a decrease in the value of the limiting current for a cation-exchange CMX membrane in a LysHCl solution compared to a NaCl solution of the same concentration is established [13]. At the same time, the over limiting current growth for a CMX/LysHCl system is observed under significantly less potential drops, which indicates more intensive development of the coupled effects of concentration polarization such as the generation of H^+^ and OH^−^ ions at the interface in the CMX/LysHCl system in comparison with the CMX/NaCl system. Electrodialysis of an individual solution of a mineral salt and mixed solutions of a mineral salt—alkylaromatic amino acid (phenylalanine, tyrosine), revealed that the presence of an amino acid in the system does not significantly affect the *i_lim_* value for anion-exchange membranes with strongly basic groups [14] and groups of mixed basicity [3]. According to the authors, this is due to the fact that the solution pH is close to the isoelectric point and most of the amino acid ions, which are in the bipolar (zwitter) form, do not take part in transferring the current through the membrane. After exceeding the limiting current density a change in the pH of the solution both at the interface and in the membrane phase due to water splitting leads to a recharge of bipolar ions and to higher values of current density at the same voltage in the system including amino acid in comparison with a single salt solution. However, an increase in the operating current density, despite an increase in the flow of amino acids, leads to a less efficient process in terms of current efficiency and energy consumption, which can be overcome by using segmented bipolar membranes that combine ionic transport and water splitting as shown in the study about the recovery of ethanolamine [15].

The influence of the nature of amino acids on current-voltage characteristics of membranes is shown in Refs [16,17,18]. For instance, an increase in the value of the limiting density, a decrease in the resistance of all three sections on the CVC and energy consumption for an electromembrane system with glutamic acid in comparison with aspartic acid at neutral pH are established [16]. A decrease in the length of the limiting current plateau by 1.3 times due to an increase in the contact angle of the liquid on the surface of the MK-40 membrane by 6–7° after electrodialysis of a mixed solution of phenylalanine and NaCl is shown in [17]. 

The shape of current-voltage curves (CVCs) and chronopotentiograms observed in inorganic ampholyte solutions differ from those obtained in strong electrolyte solutions, whose species do not enter the protonation-deprotonation reaction [5,12,19,20]. For example, papers [19,20] demonstrate the experimental CVCs of anion-exchange membranes in the solutions of Na_2_HPO_4_ and NaH_2_PO_4_, which have two inclined plateau regions related to different limiting currents. In the case of monosodium hydrophosphate, the first limiting current occurs when the NaH_2_PO_4_ salt diffusion to the membrane surface is saturated. The following growth of current density refers to the saturation of the proton flux when the membrane is almost completely converted from the H_2_PO_4_^−^ form into the HPO_4_^2−^ form. Thus, the difference in CVC for these inorganic ampholytes is due to the difference in the ratio between the pK_a1_ and pK_a2_ dissociation constants. 

Not only the nature of membranes and an electrolyte, but also the properties of the membrane surface determine their current-voltage characteristics. In the paper of Rubinstein and Zaltsman [21], it is shown that the high polarizability of the heterogeneous membrane in comparison with the homogeneous one leads to a lower value of the limiting current density in the case of an exclusively electrodiffusion mechanism. An increase in the limiting current density and a reduction in the plateau section on the CVC for homogeneous ion-exchange membranes in comparison with heterogeneous ones are experimentally established in Refs. [22,23,24]. By varying the fraction of the non-conductive surface, it is possible to increase the mass transfer through the heterogeneous membrane in comparison with the homogeneous one [25,26,27,28]. When studying mass transfer in the region of the limiting and over limiting current density through homogeneous membranes modified by applying an inert material to their surface, the optimal value of the fraction of the non-conductive surface is 35% [28] and 10% [27] for cation-exchange and anion-exchange membranes, respectively. 

To solve the problem of isolating an amino acid from a mixture with strong electrolytes, electrodialysis is environmentally and economically feasible. Applications for the desalting and separation of various amino acids by electrodialysis have been reported in [29,30,31,32,33,34]. Authors [29,30] recovered phenylalanine with the simultaneous successful removal of inorganic salts such as Na_2_SO_4_ and (NH_4_)_2_SO_4_ from mixture solutions. To increase the quality of the final product at the industrial synthesis of D-a-p-hydroxyphenylglycine, an electrodialytic process has been developed that allows the recovery of 85% of the amino acid with a salt content 70% lower than that of the initial mother liquor [31]. Desalination of milk casein hydrolysates by electrodialysis with recovery rates of 78–88% and 73–79% salt removal was carried out by Nakamura et al. [32]. It helped to improve the taste of casein hydrolysates caused by free amino acids. After the treatment of the combined electrodialysis and reverse osmosis process, the optimal recovery ratio of l-tryptophan from its crystallization wastewater reached 60%, and the purity of the final product of l-tryptophan reached 98% [2]. An economically viable in industry bipolar membrane electrodialysis technology was proposed to extract high-purity methionine (99.4%) from the mother liquor with a huge amount of inorganic salts (i.e., Na_2_SO_4_) [33]. The main focus of the article [34] is to highlight significant aspects of amino acid solution demineralization. It was shown that electrodialysis and electrodeionization are prospective technologies for such treatment. The influence of various factors on the desalination and on target product losses in this process is estimated. The effect of mineral ion nature on amino acid transport through the cation- and anion-exchange membranes and the following losses in the desalination process have been studied [34]. It was concluded that in order to reduce the losses of the amino acid, it is most preferable to use this mineral salt, which is a source of nitrogen, in microbiological synthesis. At the same time, the maximal values of the desalination degree can be achieved when using potassium chloride mineral salt. The importance of the pH of the amino acid containing solutions was established in [2,35,36,37]. To recover the L-phenylalanine from fermentation broth using electrodialysis, Choi J.-H. et al. [37] have proposed that the solution pH must be adjusted to alkaline conditions. During the neutral amino acid and chloride-ion desalination, it was found that at a pH close to the pI, the loss of glycine was at minimum and reached 6.1% of the feed concentration; at pH = 11.3 it was 51% [35]. If the pH of the dilute solution significantly affected the recovery ratio of amino acids, the salt removal ratio varied with current density and voltage [2,38]. In the process of ED desalination, Liu L.-F. et al. [2] have recommended to control the operating current below the limiting current density to avoid the polarization phenomenon. In [38] it has been shown that the desalting of the proline solution in the optimal area of current density ensures the obtaining of desalting degree up to 99.3% with small losses of the target product (~8%), while with a current higher than optimal the degree of desalting of solution is about 94.6%. 

The choice of membranes (functional groups, resin content, properties of membranes surface, etc.) and current modes are important to effective electromembrane desalination of amino acid solutions. The list of commercial membranes is quite limited. Therefore, modern research is aimed at improving their properties through the modification or minor changes in manufacturing technology. The selective and transport characteristics of membranes can be optimized by varying their quantitative composition (ion-exchange resin and inert binder) and physical parameters (resin particle size, membrane thickness and porosity, etc.). The aim of this work was to study the effect of the content of sulfonated cation-exchange resin in heterogeneous membranes on its current-voltage characteristics in mixed solutions of a mineral salt and an amino acid. This is necessary to improve the electromembrane process of demineralization of amino acids by directional selection of new membranes and current modes.

## 2. Experimental

### 2.1. Membranes

The objects of study were experimental heterogeneous ion-exchange membranes with different resin/inert binder ratios. The membranes were made from a sulfonated cation-exchanger (from 45 to 65 wt%) or a highly basic anion-exchanger (65 wt%), polyethylene binder and polyester reinforcing fabric. Auxiliary membranes used in the electrodialysis cell are the commercially available cation-exchange membrane MK-40 (LLC “Innovative Enterprise “Shchekinoazot”, Pervomayskiy, Shchekino District, Tula Region, Russia), containing a strongly acidic resin KU-2 (65 wt%), and an anion-exchange membrane MA-41 (Russia, LLC “Innovative Enterprise “Shchekinoazot”) based on a strongly basic anion-exchanger AV-17 (65 wt%) [39].

**Figure 1:**
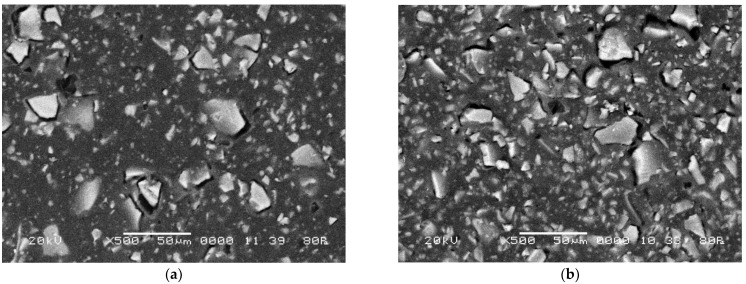
SEM images of the surface of swollen cation-exchange membranes at a magnification of 500. Ion-exchanger fraction: 45 (**a**), and 65 (**b**) wt%.

Micrographs in Figure 1 display the general appearance of a surface morphology of the membranes under study. We distinguished the polyethylene binder from functionalized ion-exchangers. There are light areas of ion-exchanger particles and dark areas of inert polymer.

**Figure 2:**
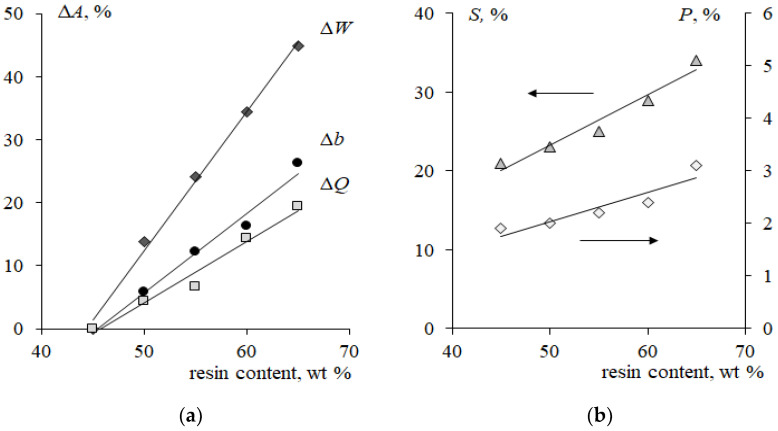
Relative changes in physical-chemical characteristics of cation-exchange membranes with different fractions of ion-exchanger (**a**) and their structural characteristics on the membrane surface (**b**). Relative changes Δ*A*% in physical-chemical parameters *A* were calculated according to the equation: Δ*A*% = 100 (*A* − *A*_45%_)/*A*_45%_, where *A*_45%_—value of a physical-chemical characteristic of the membrane with the ion-exchanger fraction of 45 wt%. *Q*—total exchange capacity per gram of the swollen membrane, mmol/g; *W*—water content, g_H_2_O_/g_swoll.membr_, %; *b*—swollen membrane thickness, μm; *S*—the fraction of the ion-exchange component, %; *P*—the fraction of macropores and structure defects, %.

An increase in the ion-exchange resin content from 45 to 65 wt% causes an increase in its fraction and porosity on the membrane surface by more than 60% (Figure 2b). Because the milling of the ion-exchanger was the same, the size of ion-exchange particles on the surface of the membrane samples remained almost constant and varied in the range from 1 to 38 μm.

For samples of the cation-exchange membrane with a minimum resin content, the total exchange capacity *Q* = 1.81 ± 0.06 mmol/g, water content *W* = 29 ± 2%, and thickness *b* = 518 ± 6 µm. With an increase in the content of sulfocation-exchange resin from 45 to 65 wt%, the total exchange capacity of the swollen membranes changed by 19% (Figure 2a). At the same time, an increase in water content and membrane thickness by 45 and 26%, respectively, was revealed.

### 2.2. Electromembrane System

**Figure 3:**
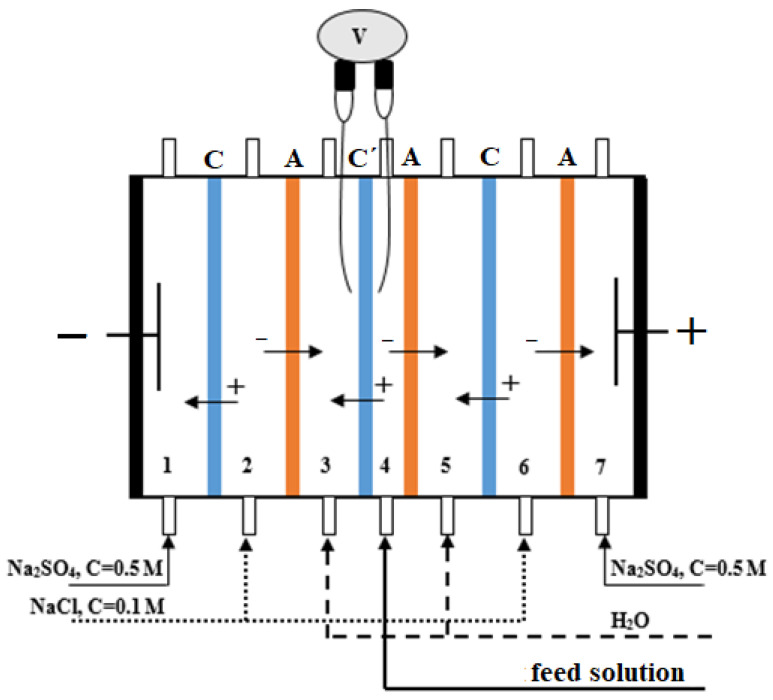
Schematic representation of the electrodialyzer with alternating cation-exchange membranes MK-40 (C) and anion-exchange membranes MA-41 (A). C′—cation-exchange membrane under study. *1*–*7*—section numbers. V—digital multimeter.

A laboratory scale seven-compartment electrodialysis cell with alternating cation- and anion-exchange membranes was used for the study of the membrane CVCs. The demineralization section consisted of an experimental membrane with different mass fractions of sulfonated cation-exchange resins and an anion-exchange membrane with a resin content of 65 wt%. The schematic configuration of the electrodialysis cell is shown in Figure 3.

The electrodialysis set-up was equipped with a power supply (Model B5-50; 0–299 V; 0.001–0.299 A) from A.S. Popov Communications Equipment Plant, JSC (Nizhny Novgorod, Russia). The experiments were carried out at constant current density and a flow rate in central and adjacent compartments of 3.5 × 10^−4^ m/s. The linear flow rate of the solutions in the electrode (*1*, *7*) and buffer (*2*, *6*) compartments was five times higher. The effective membrane area is 7.35 × 10^−4^ m^2^. The intermembrane distance in the central test section was 2 × 10^−3^ m. The height of the electrodialyzer channel was 4.2 × 10^−2^ m.

0.01 M NaCl solution or a mixture of 0.01 M NaCl solution and 0.01 M phenylalanine (Phe) solution was pumped through the desalination compartment *4*, formed by the studied cation-exchange and an auxiliary anion-exchange membrane. Concentrate compartments *3* and *5* adjacent to it were washed with distilled water. 0.1 M NaCl solution was pumped through buffer compartments *2* and *6*. The electrode chambers were washed with 0.5 M sodium sulfate solution. All of the electrodialysis experiments were performed in the range of 23 ± 1 °C. 

The voltage across the membrane under investigation was measured between two Ag/AgCl electrodes with a digital multimeter (Model APPA 207) from APPA Technology Corporation (Taipei, Taiwan). The tips of the Ag/AgCl electrodes were located on both sides of the membrane under study at a distance of 1 mm. To analyze the current-voltage characteristics, we used the corrected potential drop Δφ’, equal to the measured value of the total potential drop minus the ohmic component [40,41]. The limiting diffusion current density *i_lim_* was determined as the point of intersection of the limiting current plateau section (II) with the current axis in coordinates *i*—*f*(Δφ´). A method for determining other characteristics of the current-voltage curve is described in [42]. To assess the process, the pH of the concentrate and diluate solutions were measured. 

### 2.3. Chemical Equilibria in the System

In this work, we used the neutral alkylaromatic amino acid phenylalanine in the L-form as the object of research. The mixed solution of phenylalanine and sodium chloride has pH = 5.6–5.8, which is close to the isoelectric point of phenylalanine pI = 5.9 [29,37]. Phenylalanine has the carboxyl and ammonium groups, whose pK are 2.59 and 9.24, respectively [37].

**Figure 4:**
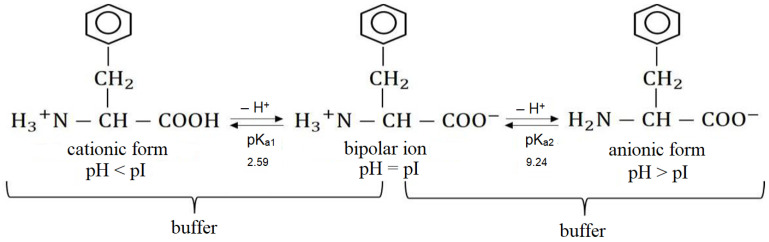
Scheme of the buffering action of phenylalanine during acidification (pH < pI) and alkalization (pH > pI) of the solution.

In solution, the equilibrium concentrations of various ionic forms of phenylalanine are determined by the protonation-deprotonation reactions (Figure 4).

The pH value of the initial mixed solution was 5.60–5.80, which corresponded to the amino acid content of 99.882–99.904% in the form of a bipolar ion, 0.060–0.095% in the cationic form and 0.023–0.036% in the anionic form. The existence of a mixture of two conjugated forms of the amino acid such as “bipolar ion/cation” or “bipolar ion/anion” is the cause of phenylalanine’s buffering properties (Figure 2). The maximum value of the buffering action is found at pH values close to their pK values. The ranges of the buffer capacity regions of the phenylalanine solutions are (2.59 ± 1) and (9.24 ± 1), respectively. 

The concentrations of Phe^+^ cations, Phe^−^ anions, and bipolar ions Phe^±^ of phenylalanine in solution are determined by the ratios [8]:

(1) $$C({\mathrm{Phe}}^{+})=\frac{C_{0}(\mathrm{Phe})}{\frac{K_{1}K_{2}}{C^{2}(H^{+})}+\frac{K_{1}}{C(H^{+})}+1}$$

(2) $$C({\mathrm{Phe}}^{-})=\frac{K_{1}K_{2}C({\mathrm{Phe}}^{+})}{C^{2}(H^{+})}$$

(3) $$C({\mathrm{Phe}}^{\pm })=\frac{K_{1}C({\mathrm{Phe}}^{+})}{C(H^{+})}$$

where $C_{0}(\mathrm{Phe})=C({\mathrm{Phe}}^{\pm })+C({\mathrm{Phe}}^{+})+C({\mathrm{Phe}}^{-})$ is the total concentration of all the phenylalanine species in solution.

**Figure 5:**
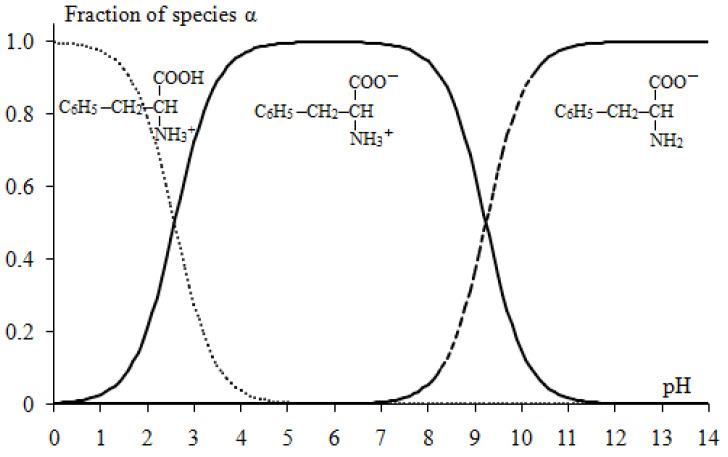
Molar fractions of the phenylalanine species vs. pH.

The distribution of phenylalanine species as a function of pH is shown in Figure 5. 

### 2.4. Physical-Chemical Properties

Prior to any measurement, we conditioned the new samples in order to stabilize their physical-chemical properties and remove impurities that may come from their manufacturing process. This treatment and the determination of the physical-chemical properties of the membranes was carried out according to standard test methods for ion-exchange membranes [43]. The total static exchange capacity of the membranes (*Q*) was determined by acid-base titration. The water content (*W*) represented the ratio of the mass of water in the swollen sample to its mass. The thickness of the samples (*b*) was measured with a micrometer. 

### 2.5. Scanning Electron Microscopy

The studies of the surface morphology of the swollen membranes were carried out by low vacuum scanning electron microscopy (SEM) using a JSM-6380 LV microscope (JEOL Ltd., Tokyo, Japan). The quantitative estimation of fraction of ion-exchangers and macropores was carried out with the help of the authors’ software by using the digital processing of SEM images [44]. The fraction of the ion-exchanger (macropores) was determined as the ratio of the total area of the ion-exchanger (macropores) to the area of the scanned area.

## 3. Results and Discussion 

### 3.1. Influence of Phenylalanine on CVCs of Sulfonated Cation-Exchange Membranes

**Figure 6:**
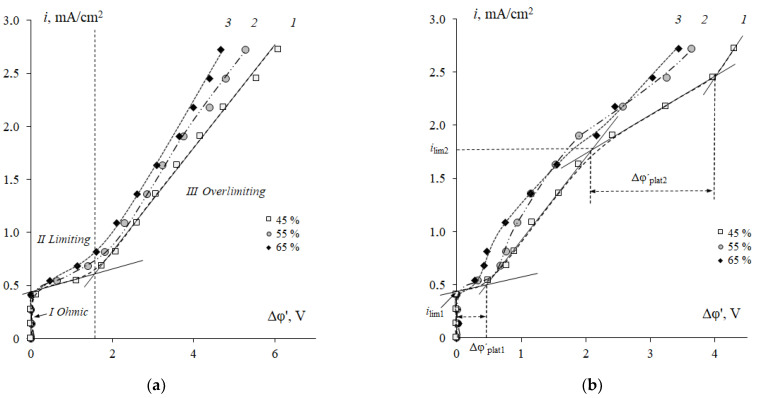
CVCs of cation-exchange membranes with the resin content of 45 (*1*), 55 (*2*) and 65 (*3*) wt% in 0.01 M NaCl solution (**a**) and in the mixed 0.01 M NaCl + 0.05 M Phe solution (**b**). Δφ´—corrected potential drop.

Figure 6 presents a comparison of the current-voltage characteristics of cation-exchange membrane samples with a different ratio of resin/polyethylene in 0.01 M NaCl solution or in 0.05 M Phe + 0.01 M NaCl solution.

In the case of the strong electrolyte solution (Figure 5), the shape of the experimental curves is close to those described in the Refs. [42,45,46,47]. The initial part (I) of the CVC, approximately at *i* < *i_lim_*, is linear in *i*—Δφ coordinates. Then there is an inclined region of limiting current plateau (II) starting at *i* ≈ *i*_lim1_. At currents *i > i*_lim1_, there are the process of water splitting at the membrane/solution interface, the transfer of H^+^ ions to the concentrate compartment, and the accumulation of OH^−^ ions at the surface of cation-exchange membranes [48]. This region of currents is characterized by the stable electroconvection regime [23,49]. The region of the secondary increase in the current (III) is the part related to the over limiting current density, where oscillations of the potential drop are observed. These oscillations occur due to the generation of unstable electroconvective vortices at the interface [26,49,50,51]. 

The shape of CVCs of cation-exchange membranes observed in the mixed NaCl + Phe solution (Figure 6b) generally differs from those obtained in the NaCl solution (Figure 6a). An initial section (I) is linear, as in the case of the NaCl solution. Section I (*i* < *i_lim_*) corresponds to the stage when sodium cations are the main current carriers in the electromembrane system. In this current range, insignificant transport of phenylalanine through the cation-exchange membrane occurs due to diffusion transfer and due to the electroosmotic mechanism (conjugated transfer of amino acids in the hydration shell of sodium ions). Electromigration of amino acid cations is small, since the Phe^+^ ion content in the feed solution is 0.06%, which corresponds to *C*_0_(Phe^+^) = 3 × 10^−5^ M. In this regard, the presence of amino acid in sodium chloride solution at *C*_0_(Phe) = 5 × *C*_0_ (NaCl) insignificantly affects the value of the limiting diffusion current density on the cation-exchange membrane. The *i*_lim1_ value corresponds to the limiting diffusion current density for sodium cations.

In an aqueous-salt solution of phenylalanine, the main differences in the parameters of the current-voltage curve of membranes in comparison with the sodium chloride solution were established in regions II (limiting) and III (underlimiting) of the CVCs (Figure 6b). First, a significant decrease in the length of the plateau of the limiting current for the ion Na^+^ and a decrease in the resistance *R*_2_ of the second section were established. For a membrane with the cation-exchange resin content of 45 wt%, a decrease in the length of the plateau section of the limiting current by 60% and the resistance *R*_2_ by 50% was revealed (Table 1). 

At *i* = *i*_lim1_, the concentration of the main current carriers such as Na^+^ cations at the membrane surface can decrease to values comparable to the concentration of H^+^ (OHˉ) ions formed as a result of water splitting. According to [52,53], phenylalanine, like any organic acid, is a catalyst for the water splitting reaction and contributes to an increase in the number of additional current carriers. In addition, at *i* = *i_lim_*, as a result of protolytic reactions (2), bipolar amino acid ions are recharged into anions at the membrane/solution boundary (barrier effect [1]) with the formation of protons, which are also involved in the transfer through the membrane. The lower value of the resistance *R*_2_ in mixed solutions may be caused by a partial conversion of the cation-exchange membrane to the hydrogen form. Thus, the fraction of the current carried by the Phe^+^ cations increases. Moreover, additional current carriers appear. They are H^+^ ions that are products of water splitting. Another reason for the decrease in *R*_2_ resistance is the hydrophobization of the cation-exchange membrane surface, which creates conditions for the development of equilibrium electroconvection in a stable regime, similar to the Dukhin-Mishchuk regime [54]. The change in the hydrophilic-hydrophobic balance of the membrane surface occurs due to the adsorption of the aromatic amino acid with hydrophobic properties [55]. The reduction in the plateau length is evidence that the unstable electroconvection regime (Rubinstein-Zaltzman regime) occurs at a smaller value of the potential drop. The transition to the unstable regime of electroconvection corresponds to the region of the secondary increase in the current (III) of the CVC. With an increase in the hydrophobic properties of the membrane surface, the intensity of unstable electroconvective mixing of the solution, arising as a result of the effect of an electric field on the space electric charge in a depleted solution at the boundary with the membrane, increases [56,57,58]. Therefore, the resistance *R*_3_ of the third region on the current-voltage curve decreases by 42% in the presence of phenylalanine.

The main specific difference is that in mixed solutions, the CVCs have two inclined plateau sections (II and IV), while in the strong electrolyte solution, only one section (II) is established. The CVCs show the presence of *i*_lim2_, the values of which exceed the *i*_lim1_ values. A similar phenomenon was found for anion-exchange membranes in mineral ampholyte solutions [20]. However, such an effect has not been reported in any of the known works devoted to the study of the CVCs of ion-exchange membranes during the electrodialysis of amino acid containing solutions. 

### 3.2. Features of the H^+^/OH^−^ Ion Generation in the Electromembrane System with Cation-Exchange Membranes and the Neutral Amino Acid

**Figure 7:**
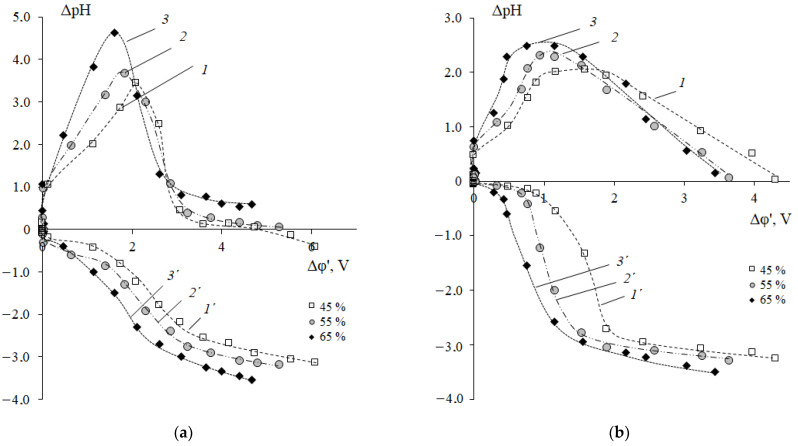
Dependences of the ΔpH of the solution at the outlet and inlet of the demineralization (curves 1–3) and concentrate (curves 1´–3´) compartments on the corrected potential drop Δφ´ during electrodialysis of 0.01 M NaCl solution (**a**) and the mixed 0.01 M NaCl + 0.05 M Phe solution (**b**). Cation-exchange membranes with the resin content of 45 (*1*, *1´*), 55 (*2*, *2´*) and 65 (*3*, *3´*) wt%.

Figure 7 shows the pH difference at the inlet and outlet of the desalination and concentrate compartments. ΔpH is the result of the interaction of the fluxes of H^+^ and OH^−^ ions entering the chamber solution from the membrane surface. If the solution of the desalination compartment is alkalized and the value of ΔpH > 0, then water splitting is more intense at the cation-exchange membrane interface. In the case of acidification of the solution in the desalination compartment (ΔpH < 0), water splitting is more intense at the anion-exchange membrane interface. An increase in the pH of the solution in the demineralization compartment and acidification of the solution in the concentrate compartment (Figure 7a) correspond to the region of the inclined section of the limiting current plateau (II) on the CVCs of membranes in 0.01 M NaCl solutions.

The maxima on the curves of the dependence of ΔpH on the reduced potential drop and the subsequent decrease in the value of ΔpH of the solution in the demineralization compartment (Figure 7a, curves 1–3) to the initial values are established both in 0.01 M NaCl solution and in the mixed 0.01 M NaCl + 0.05 M Phe solution. This is explained by the fact that when the limiting current density on the anion-exchange membrane reaches *i_lim_* = 0.65 mA/cm^2^, the process of water splitting begins. The experimental cation-exchange membranes contain functional sulfogroups, the catalytic activity of which is considered to be very weak with respect to the water splitting (the rate constant of the water splitting reaction is 10^−3^ s^−1^) [59]. This value is greater than the catalytic activity of quaternary amino groups (*k_lim_* ~ 0 s^−1^), which are part of the used highly basic anion-exchange membrane [59]. However, under intense current conditions in the surface layer of anion-exchange membranes, partial hydrolysis of strongly basic ionogenic groups (quaternary ammonium bases) can occur with the formation of fixed tertiary and secondary amino groups, which are catalytically active in the water splitting reaction [60,61]. Therefore, the amount of H^+^ ions entering the demineralized solution from the surface of the anion-exchange membrane is greater than the amount of OH^−^ ions delivered from the cation-exchange membrane. As a result, acidification of the solution to the values corresponding to the initial solution is observed in the demineralization compartment (Figure 7, curves 1–3). Maximum change in pH at the outlet and inlet of the desalination compartment during electrodialysis of the 0.01 M NaCl solution reaches a value of 4.62. This is two times higher than the corresponding value for the mixed 0.01 M NaCl + 0.05 M Phe solution. Smaller ΔpH in the demineralization compartment during electrodialysis of the mixed NaCl + Phe solution is due to the buffering effect because of the participation of phenylalanine in the protonation–deprotonation reactions (Figure 4). 

**Figure 8:**
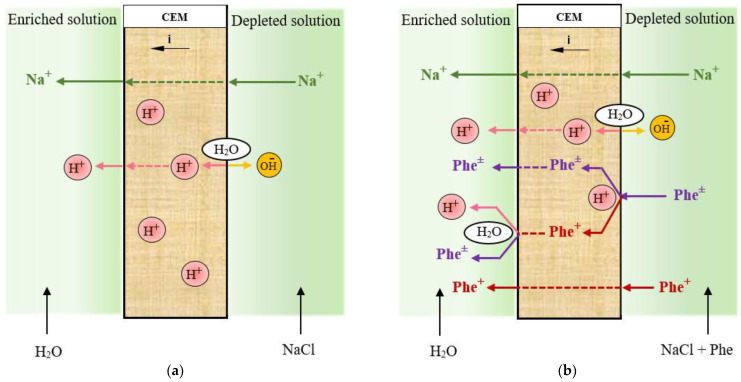
A schematic representation of ion transport, water splitting and aside dissociation phenomena in the systems CEM/NaCl solution (**a**) and CEM/NaCl + Phe solution (**b**).

In the solution of the concentrate compartment, stronger acidification was revealed in the case of the mixed 0.01 M NaCl + 0.05 M Phe solutions. In this case, at the solution/membrane interface, two processes of generation of H^+^ and OH^−^ ions run in parallel: with the participation of the cation-exchange membrane functional groups in the demineralization compartment and due to protolytic reactions of the amino acid in the solution of the concentrate compartment. The Donnan exclusion of OH^−^ ions (as co-ions) from a cation-exchange membrane leads to the fact that pH of the internal membrane solution is smaller than that of the external bulk solution [62]. Therefore, the ratio of the Phe^+^ cation concentration to the bipolar Phe^±^ ion or Phe^−^ anion concentration is higher in the cation-exchange membrane than in the external solution. The second mechanism, where H^+^ ions are generated during acid dissociation at the depleted solution/ion-exchange membrane interface, is well understood for anion-exchange membrane systems with polybasic acid salt solutions [63]. The “acid dissociation” mechanism is typical only for ampholytes, the electric charge of which depends on the pH of their solutions. We have shown for the first time the possibility of implementing this mechanism for the generation of H^+^/OH^−^ ions at the enriched solution/cation-exchange membrane interface during electrodialysis of amino acid containing solutions. Figure 8 shows a schematic representation of H^+^/OH^−^ ion generation in the systems’ CEM/NaCl solution and CEM/NaCl + Phe solution. Phe^+^ cations are contained in the demineralized solution, and they are additionally formed because of the recharging of bipolar phenylalanine ions in the highly acidic medium of the membrane phase. After being transferred through the cation-exchange membrane (with a lower pH of the internal membrane solution) to the solution (with a higher pH) of the concentrate compartment, amino acid cations transformed into the bipolar form (Figure 4). Formed H^+^ ions are transported by the electric field from the interface into the bulk solution and enhance the acidification effect of the solution in the concentrate section compared to a system containing only mineral salt.

**Figure 9:**
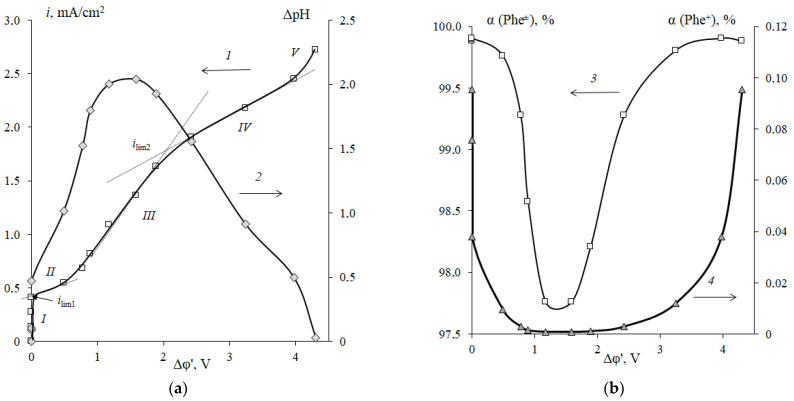
The CVC (*1*) and dependences of ΔpH (*2*) of the solution at the inlet and outlet of the demineralization compartment (**a**), the fraction of bipolar ions (*3*) and cations (*4*) of phenylalanine in the demineralized solution (**b**) on the corrected potential drop on the membrane (with resin content of 45 wt%) during electrodialysis of the mixed NaCl + Phe solution.

Figure 9 shows a comparative analysis of the CVC of the cation-exchange membrane in 0.01 M NaCl + 0.05 M Phe solution, changes in the pH of the demineralized solution and the corresponding values of the fractions of the amino acid in various ionic forms, calculated according to (1–3). The first inclined plateau and the *i*_lim1_ value correspond to the state of the system when the concentration of the main carriers, which are sodium cations, in the solution at the membrane surface is much lower than their concentration in the bulk of the demineralized solution. This state corresponds to the maximum flow of the amino acid through the membrane [1]. The reason for the appearance of the second sloping plateau on the current-voltage curve is the ability of phenylalanine as an organic ampholyte to participate in protolytic reactions. A significant change in the pH of the solution in the demineralization compartment affects the quantitative ratio of different types of current carriers in the solution at *i* > *i_lim_* (Figure 9a). An increase in the amount of OHˉ ions at the membrane/solution interface participating in the recharge of the amino acid with the formation of anions with an increase in the current leads to a decrease in the content of bipolar Phe^±^ ions and Phe^+^ cations in the demineralized solution participating in the current transfer through the membrane (Figure 9b). The appearance of the second section of the limiting current plateau (IV) on the CVC corresponds to the minimum content of both Phe^+^ cations and bipolar Phe^±^ ions in the solution of the demineralization compartment. In the region of the limiting current density *i*_lim2_, the fraction of the amino acid in the cation form is negligible. For the system with a membrane containing 45 wt% of ion-exchange resin, the concentration of Phe^+^ cations in the solution is (5–6) × 10^−7^ M at a total concentration of the amino acid *C*_0_(Phe) = 0.05 M. Further acidification of the demineralized solution due to the reaction of water splitting at the anion-exchange membrane interface leads to an increase in the concentration of amino acid cations involved in the transfer through the membrane. 

### 3.3. Influence of Resin Content on CVCs of Cation-Exchange Membranes in Strong Electrolyte and Phenylalanine-Containing Solutions 

**Figure 10:**
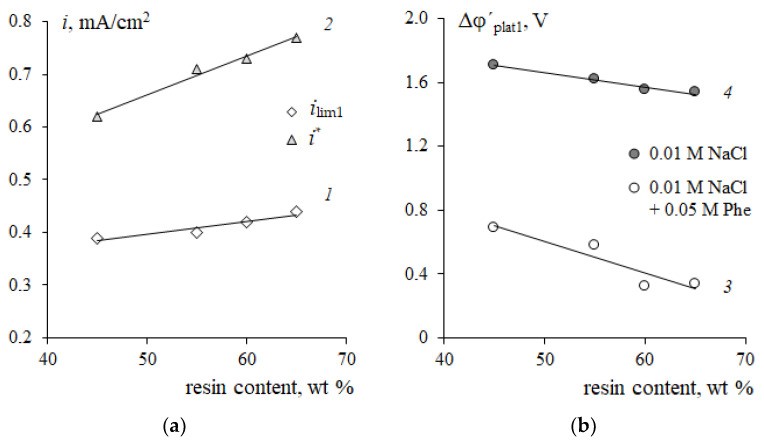
Dependences of the limiting current *i*_lim1_ (*1*), the current of onset of unstable electroconvection i* (*2*) (**a**), and the plateau length Δφ’_plat1_ (3, 4) (**b**) on the CVCs of cation-exchange membranes in the 0.01 M NaCl solution (*1*, *2*, *4*) and in the 0.01 M NaCl + 0.05 M Phe solution (*3*) on the resin content.

Figure 6 shows the effect of the resin content in the membrane on the form and quantitative characteristics of the CVC in the NaCl solution and in the mixed NaCl + Phe solution. An increase in the resin content is accompanied by the convergence of the conducting surface zones (ion-exchanger particles), and the structure of the membrane surface becomes more uniform (Figure 1) [44]. It was shown in [22,23,64,65] that surface homogenization leads to an increase in the average limiting current density and a reduction in the length of the limiting current plateau on the CVC. In the range of changes in the resin content from 45 to 65% wt%, a significant increase in the fraction of the ion-exchanger on the membrane surface was found (Figure 2b). Therefore, an increase in the value of the limiting current *i*_lim1_ by 10% in the NaCl solution was revealed (Figure 10, curve 1). The length of the limiting current plateau characterizes the ability of the electromembrane system to develop electroconvection [66,67,68]. With an increase in the resin content, a decrease in the plateau length (II) on the CVCs was established (Figure 10, curves 3, 4). The length of the plateau Δφ´_plat1_ on the current-voltage curve of membranes the NaCl solution decreased by 10%, while in a mixed solution with phenylalanine, it decreased by 50%. The current of onset of unstable electroconvection i* increases due to a decrease in the resistance *R*_2_ of the system with an increase in the ion-exchanger content (Figure 10, curve 1). 

**Figure 11:**
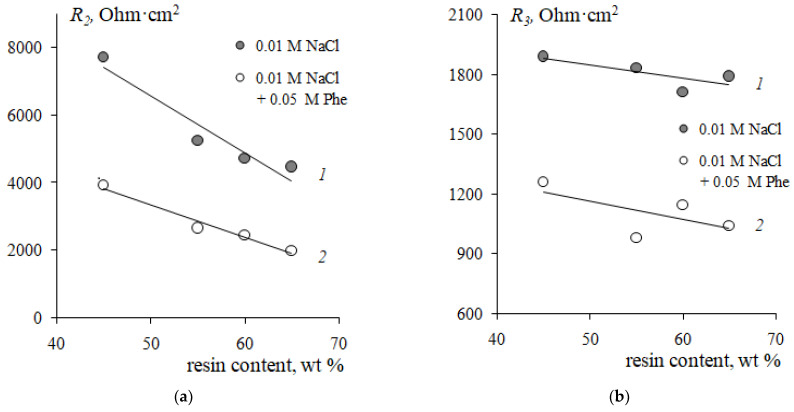
Dependences of the resistances of the second (limiting) *R*_2_ (**a**) and the third (underlimiting) *R*_3_ (**b**) sections of CVCs of cation-exchange membranes in 0.01 M NaCl solution (*1*) and the mixed 0.01 M NaCl + 0.05 M Phe solution (*2*) on the resin content.

An increase in the resin content in membranes leads to an increase in the angle of inclination of the limiting current plateau with respect to the potential axis both in the system with the mineral salt and in the mixed solution with phenylalanine (Figure 6). This corresponds to a decrease in the resistance of the electromembrane system (Figure 11a). Traditionally, a decrease in resistance in this region is associated with the appearance of additional current carriers such as H^+^ and OH^−^ ions, which are formed in the solution at the interface with the membrane because of the water splitting reaction with the participation of fixed groups. The increase in the pH value of the solution in the demineralization compartment and acidification of the solution in the concentrate compartment (Figure 7) confirms this fact. In the case of a membrane with a maximum content of ion-exchange resin, the process of water splitting is more intense compared to a membrane containing 45 wt% ion-exchange resin. The reason is the differences in the values of the total exchange capacity and the structure of the membrane surface (Figure 2). A greater number and availability of ion-exchange groups that catalyze the water splitting reaction cause a more intense generation of hydrogen and hydroxide ions in solution at the interface with the membrane. The rate of generation of hydrogen and hydroxide ions during the water splitting is determined by the different distribution of the charge density and field strength near the membrane–solution interface. The results obtained agree with the data from [23] that the water splitting is accelerated when a homogeneous membrane is used. In Refs. [49,69], the concept of a “limiting current plateau” is associated with a stable regime of electroconvection proceeding by the mechanism of electroosmosis of the first kind. An increase in the slope of the plateau corresponds to more intensive stable electroconvection. With an increase in the ion-exchanger content from 45 to 65 wt%, a decrease in the resistance of the second (limiting) section on the CVCs by 40% in the NaCl solution and by 50% in the mixed NaCl + Phe solution was revealed (Figure 11a).

A negative linear correlation was established between the value of the second limiting current *i*_lim2_ and the ion-exchange resin content in the membrane *W* (wt%): $i_{lim2}=3.51-0.0036W$ (*r*^2^ = 0.80). The reason is that with an increase in the resin content, the alkalization of the solution in the demineralization section increases (Figure 7b), and the concentration of Phe^+^ cations as carriers decreases, respectively. 

The appearance of potential oscillations in the region of the third section of the CVC (Figure 6) indicates the appearance of unstable electroconvective vortices, which facilitate more intensive delivery of new portions of the solution from the bulk to the membrane surface. This causes a decrease in the resistance of the system *R*_3_ (Figure 10b). The presence of phenylalanine promotes a more intensive electroconvective mixing of the solution at the interface and is accompanied by a more significant decrease in the resistance *R*_3_ with an increase in the ion-exchanger content as compared to the mineral salt solution. 

Thus, with an increase in the resin content in the cation-exchange membrane, a decrease in the plateau of the limiting current and a decrease in the resistance of the second and third sections on the current-voltage curve are established. The revealed changes in the CVC parameters indicate an increase in the ability of the electromembrane system to develop electroconvection with an increase in the ion-exchange resin content. The presence of the neutral amino acid in the solution leads to a significant increase in the influence of the content of the ion-exchange resin in the membrane on the revealed effects.

The results presented in Figure 10 and Figure 11 make it possible to choose the conditions for the efficient separation of a neutral amino acid and a mineral salt by electrodialysis. On the one hand, the use of a membrane with the maximum mass fraction of resin contributes to an increase in the limiting current (*i_lim_*) and a decrease in the resistance of the electromembrane system. These factors allow to predict an increase in the mass transfer of mineral ions and the degree of solution demineralization, as well as a decrease in the energy consumption of the process. On the other hand, for membranes with a high content of ion-exchange resin, a tendency was found to decrease in the potential drop corresponding to the development of an unstable electroconvection regime. The appearance of an additional electroconvective mechanism for the transport of components can lead to an increase in undesirable losses of the target component (amino acids) and a decrease in the factor of their separation. Demineralization of the solution must be accomplished with a minimum loss of the amino acid. It should also be noted that the structure of the ion-exchange membrane plays a significant role in electrodialysis. An increase in membrane porosity with an increase in the ion-exchange resin content (Figure 2b) contributes to amino acid losses. The results in Figure 7b are an additional argument that the use of a membrane with a maximum resin content has the strongest effect on the intensity of amino acid mass transfer during electrodialysis. This membrane is characterized by the maximum increase in the pH of the solution in the demineralization section at the limiting current mode and the more dramatic decrease in the pH at the over limiting mode of electrodialysis. These changes in the pH of the solution suggest a limitation of the transmembrane transfer of amino acids in the range of currents 1.0 < *i/i*_lim1_ < 2.0 and an increase in amino acid losses at *i/i*_lim1_ > 2.0 currents. If it is necessary to reach deep demineralization, the membrane with resin content of 65 wt% and an intense current regime (*i/i*_lim1_ > 2.0) are required. If it is necessary to reduce the loss of the target product (amino acid), it is necessary to carry out the process of separating the amino acid and the mineral salt at *i/i*_lim1_ ≈ 1.0 currents corresponding to the region of the barrier effect. Further studies of transport characteristics of the studied experimental membranes in a wide range of currents are required to determine the conditions for the effective separation of a neutral amino acid and a mineral salt in solution. 

## 4. Conclusions

The effect of the cation-exchange resin content in experimental heterogeneous membranes on the current-voltage characteristics during electrodialysis of the NaCl solution and the mixed NaCl + Phe solution was established. This manifests itself in a decrease in the length of the plateau of the limiting current and in the resistances of all sections of the CVC with an increase in the mass fraction of the ion-exchanger in the membrane. The observed effects are due to the fact that the resin content in the membrane affects both the intensity of the reaction of water splitting at the interface and the development of electroconvection in intensive current modes. 

The effect of the amino acid on the CVCs of all experimental membrane samples was established. It was shown that the specific shape and quantitative changes in the current-voltage characteristics of experimental membrane samples in aqueous salt solutions of phenylalanine are determined by two factors. The first one is the ability of the amino acid to recharge when the pH of the solution changes is the reason for the appearance of the second plateau of the limiting current. For the first time, the possibility of the generation of H^+^/OH^−^ ions through acid dissociation at the enriched solution/cation-exchange membrane interface is shown in the electromembrane system with the phenylalanine containing solution. The second factor is the change in the hydrophilic-hydrophobic balance of the membrane surface after contact with the aromatic amino acid that causes a decrease in the length and resistance of the limiting current plateau, as well as a decrease in the resistance of sections corresponding to a further increase in the current. It was shown that the presence of the amino acid also enhances electroconvection due to the participation of phenylalanine in protolytic reactions, which leads to a decrease in changes in the pH of the solution in the demineralization compartment. An increase in the resin content in the membrane from 45 to 65% during electrodialysis of the NaCl solution causes a shortening of the first plateau section of the limiting current Δφ´_plat1_ by 10%, and for the mixed NaCl + Phe solution by 50%. 

The results of this study make it possible to make a preliminary selection of membranes and current modes for the efficient separation of neutral amino acids and mineral salts by electrodialysis.

## Figures and Tables

**Table 1:** Parameters of the CVCs of the cation-exchange membrane with a resin content of 45% in 0.01 M NaCl solution and the mixed 0.01 M NaCl + 0.05 M Phe solution.

NaCl				NaCl + Phe				
*i*_lim1_, mA/cm^2^	Δφ´_plat1_, V	*R*_2_, Ohm∙cm^2^	*R*_3_, Ohm∙cm^2^	*i*_lim1_, mA/cm^2^	*i*_lim2_, mA/cm^2^	Δφ´_plat1_, V	*R*_2_, Ohm∙cm^2^	*R*_3_, Ohm∙cm^2^
0.39	1.71	7730	1890	0.40	1.76	0.69	3930	1260

## Data Availability

Data is contained within the article.

## Nomenclature

*b*—swollen membrane thickness; *C*—concentration; *i*—current density; *i_lim_*—limiting current density; *i*_*lim*1_—first limiting current density; *i*_*lim*2_—second limiting current density; *P*—the fraction of macropores and structure defects; Phe—phenylalanine; pI—isoelectric point; pK—index to express the acidity of weak acids protonation-deprotonation reaction; *R*_2_—resistance of the second section on CVC; *R*_3_—resistance of the third section on CVC; *Q*—total exchange capacity; *S*—the fraction of the ion-exchange component; *W*—water content; α—molar fraction of species; Δφ—total potential drop; Δφ’—corrected potential drop; Δφ´_plat_—inclined plateau length; CVC—current-voltage curve; SEM—scanning electron microscopy.
